# NK cell subsets define sustained remission in rheumatoid arthritis

**DOI:** 10.1172/jci.insight.182390

**Published:** 2024-12-06

**Authors:** Carl Coyle, Margaret Ma, Yann Abraham, Christopher B. Mahony, Kathryn Steel, Catherine Simpson, Nadia Guerra, Adam P. Croft, Stephen Rapecki, Andrew Cope, Rowann Bowcutt, Esperanza Perucha

**Affiliations:** 1 Centre for Inflammation Biology and Cancer Immunology, Floor 1, New Hunt’s House, Great Maze Pond, King’s College London, Guy’s Campus, London, United Kingdom.; 2 Centre for Rheumatic Diseases, King’s College London, London, United Kingdom.; 3 Level 10, Tower Block, Division of Rheumatology, University Medicine Cluster, National University Health System, Singapore.; 4 Department of Medicine, National University Singapore, Singapore.; 5 Janssen Pharmaceuticals, Turnhoutseweg, Belgium.; 6 Rheumatology Research Group, Institute of Inflammation and Ageing, Queen Elizabeth Hospital, and; 7 Birmingham NIHR Biomedical Research Centre, University of Birmingham, Birmingham, United Kingdom.; 8 UCB Biopharma, Slough, London, United Kingdom.; 9 Faculty of Natural Sciences, Department of Life Sciences, Imperial College London, London, United Kingdom.

**Keywords:** Autoimmunity, NK cells, Rheumatology, Tolerance

## Abstract

Rheumatoid arthritis (RA) is an immune-mediated, chronic inflammatory condition. With modern therapeutics and evidence-based management strategies, achieving sustained remission is increasingly common. To prevent complications associated with prolonged use of immunosuppressants, drug tapering or withdrawal is recommended. However, due to the lack of tools that define immunological remission, disease flares are frequent, highlighting the need for a more precision medicine–based approach. Utilizing high-dimensional phenotyping platforms, we set out to define peripheral blood immunological signatures of sustained remission in RA. We identified that CD8^+^CD57^+^KIR2DL1^+^ NK cells are associated with sustained remission. Functional studies uncovered an NK cell subset characterized by normal degranulation responses and reduced proinflammatory cytokine expression, which was elevated in sustained remission. Furthermore, flow cytometric analysis of NK cells from synovial fluid combined with interrogation of a publicly available single-cell RNA-Seq dataset of synovial tissue from active RA identified a deficiency of the phenotypic characteristics associated with this NK cell remission signature. In summary, we have uncovered an immune signature of RA remission associated with compositional changes in NK cell phenotype and function that has implications for understanding the effect of sustained remission on host immunity and distinct features that may define operational tolerance in RA.

## Abstract 2

Distinct NK cell subsets in peripheral blood characterise the immune system of RA patients achieving long-term remission

## Graphical abstract

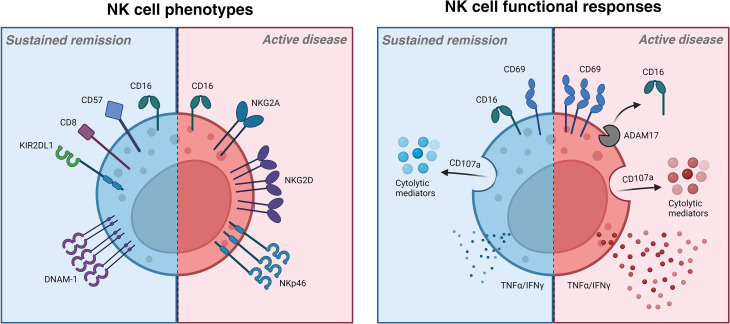

## Introduction

Driven by the introduction of more targeted therapeutics and improved clinical management strategies, obtaining clinical remission in patients with rheumatoid arthritis (RA) is now a realistic goal (1). Owing to the complications associated with prolonged immunosuppression (2), clinical management of RA has shifted focus toward optimizing strategies for drug dose tapering or, in favorable circumstances, complete drug withdrawal. Despite this ambitious goal, multiple studies to date have described that, in the context of drug tapering/withdrawal, disease flare occurs in 40%–50% of patients (3). While this highlights that achieving drug-free remission is possible in a subset of patients, the tools to identify these patients are lacking.

Analysis of clinical parameters suggests that lower baseline disease activity and evidence of sustained remission prior to drug tapering is associated with obtaining drug-free remission (4). Therefore, the European Alliance of Associations for Rheumatology recommendations indicate that drug dose tapering should only be considered in patients who demonstrate sustained remission (5). However, evidence shows that residual disease may still be present in some patients achieving clinical remission by even the most stringent criteria (6). Within a subset of patients who achieve sustained clinical remission, residual inflammatory disease will increase the risk of disease flare upon treatment cessation. The failure of clinical disease activity scores to capture the underlying biological heterogenicity of the remission state underscores the urgent need to redefine clinical remission at a molecular and cellular level to uncover biomarkers of sustained deep remission. Such biomarkers could contribute precision medicine approaches in RA routine management.

Studies examining the immunological landscape of sustained clinical remission are limited. However, with the expansion of high-dimensional single-cell technology combined with multi-omics approaches, data are beginning to emerge (7–12). Integrated results from histological analysis and RNA-Seq data have highlighted the heterogeneity of the RA synovial pathotypes and prompted investigations into whether differences in synovial architecture are associated with treatment response and long-term patient outcomes (8, 13). For example, single-cell transcriptomic analysis has revealed specific immune subsets associated with sustained remission such as MerTK^+^CD206^+^ macrophages, which promote repair processes in fibroblast-like synoviocytes (7). A recent study identified by mass cytometry that cytotoxic and exhausted CD4^+^ memory T cells, memory CD8^+^CXCR5^+^ T cells, and IGHA1^+^ plasma cells were associated with disease flares upon treatment cessation (9).

To uncover potentially novel immune signatures of the sustained remission state, we undertook an unbiased, cell-agnostic approach, exploiting high-dimensional phenotyping technology to profile the immune landscape of patients with RA in clinically defined sustained remission derived from the REMission in RA (REMIRA) cohort (14). Our aim was to define remission signatures in the context of peripheral blood, as the minimally invasive nature of obtaining such samples should prove advantageous to the feasibility of translating such signatures into clinical practice. Through this approach, we have identified key phenotypic and functional subsets of NK cells as immune signatures of sustained RA remission. These signatures provide insight into the effect of sustained remission on host defence systems, facilitating the assessment of operational tolerance in RA.

## Results

### Peripheral blood CD8^+^CD57^+^ NK cells are associated with sustained RA remission.

**Figure 1:**
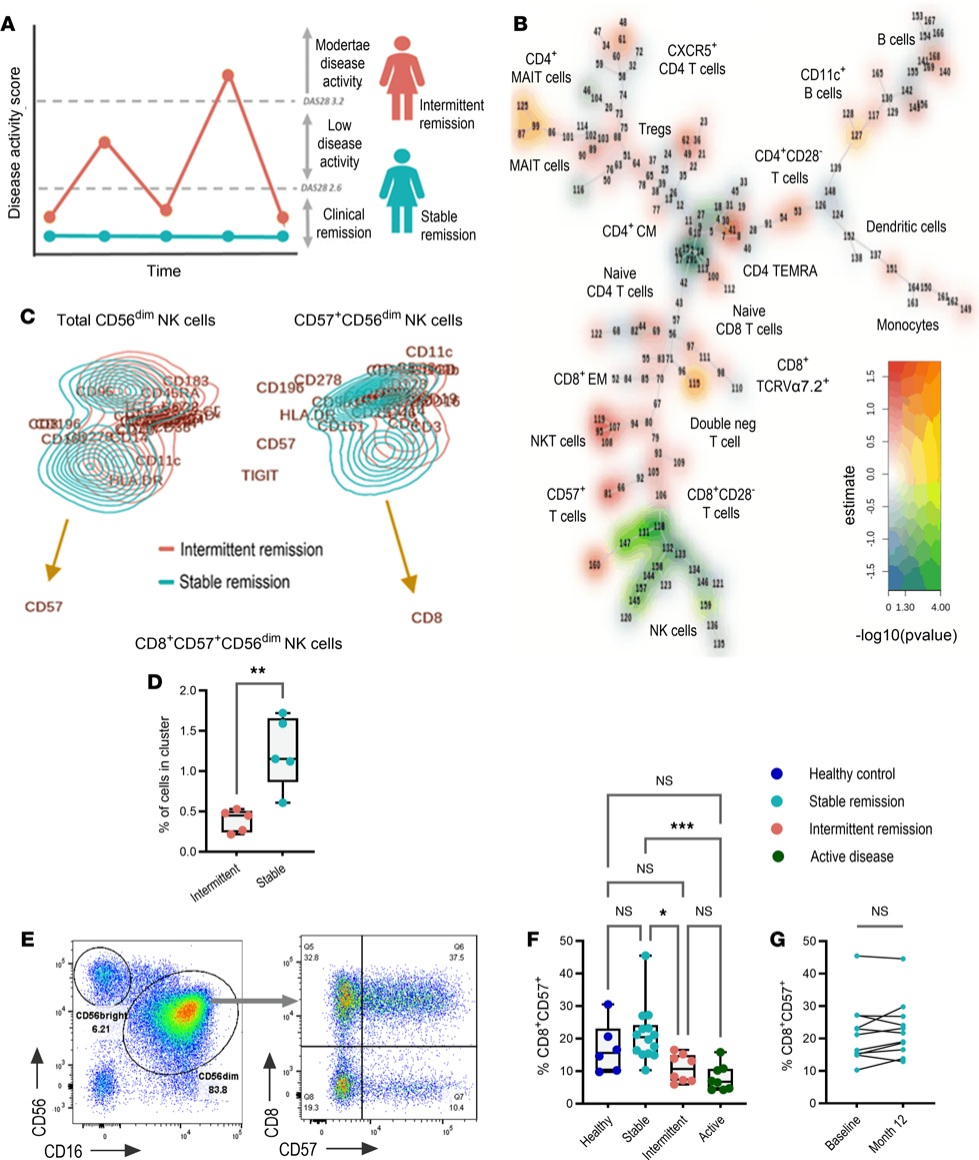
Peripheral blood CD8^+^CD57^+^ NK cells are associated with sustained RA remission. (**A**) Longitudinal changes in DAS28 scores define key patient trajectory groups. (**B**) Multiple spanning tree visualization depicting differential cluster abundance analysis (comparing intermittent to stable remission) according to differences in fold change (color gradient along the *y* axis) and in order of increasing significance (color gradient along the *x* axis). (**C**) FreeViz analysis comparing total CD56^dim^ NK cells (left) and total CD56^dim^CD57^+^ NK cells (right). (**D**) The percentage of cells in CD8^+^CD57^+^ NK cell clusters between intermittent (*n* = 5) and stable remission (*n* = 5). (**E**) Flow cytometry gating to define CD56^dim^ NK cells (left) followed by subsetting based on CD8 and CD57 expression (right). (**F**) The proportion of CD8^+^CD57^+^ CD56^dim^ NK cells across healthy donors (*n* = 6), stable remission (*n* = 14), intermittent remission (*n* = 8), and active disease (*n* = 8). (**G**) The proportion of CD8^+^CD57^+^ NK cells from paired baseline and month 12 samples from stable RA remission (*n* = 10). Whiskers on plots represent minimum to maximum values. *P* values were determined by using the Mann-Whitney *U* test (**D**), Kruskal-Wallis test with Dunn’s multiple-test correction (**F**), and Wilcoxon matched-pairs signed-rank test (**G**). *P < 0.05, **P < 0.01, ***P < 0.001.

To define immune signatures of sustained RA remission, longitudinal changes in DAS28-CRP scores from the REMIRA cohort (14) were scrutinized to define differential patient trajectory groups. Individuals who maintained DAS28 remission for a 12-month period were denoted as “stable remission” while individuals who cycled between periods of remission and low to moderate disease activity, suggestive of unstable disease control, were denoted as “intermittent remission” (Figure 1A). To investigate changes in the immune system between remission groups, samples were selected from 5 patients from both groups at time points where DAS28 < 2.6 (indicating clinical remission; longitudinal DAS28 scores and selected samples from both groups are detailed in Table 1) and stained for mass cytometry immune phenotyping (panel information is detailed in Supplemental Table 1; supplemental material available online with this article; https://doi.org/10.1172/jci.insight.182390DS1). Matching samples according to DAS28 remission scores allowed us to compare stable and intermittent remission groups at a time when both were deemed to be in clinical remission, despite longitudinally progressing on 2 very different clinical trajectories. A summary of participants’ demographics, clinical information, and treatments are provided in Table 2.

Cells with similar phenotypes were automatically grouped using FlowSOM (15), generating 169 clusters, which were manually annotated into metaclusters. A generalized linear mixed model was fit to the data to assess differential cluster abundance between intermittent and stable remission, results of which were integrated into a minimal spanning tree visualization plot (Figure 1B). While results show fold-change differences in multiple clusters, a collection of clusters corresponding to CD56^dim^ NK cell subsets were found to be significantly depleted, indicating an increased proportion in these populations in stable remission when compared with intermittent remission.

To identify compositional shifts within CD56^dim^ NK cell subsets between remission groups and identify surface markers that drive these differences, we utilized FreeViz analysis (16). Firstly, the data identify that expression intensity of CD57 on CD56^dim^ NK cells was higher in stable remission when compared with intermittent remission (Figure 1C). Given that CD57 expression is associated with NK cell maturation (17) our data suggest that increased expression of markers of mature NK cell subsets were associated with stable RA remission. Secondly, FreeViz analysis of the CD57^+^CD56^dim^ NK cell subset demonstrated that expression of CD8 contributed further to the separation of mature CD57^+^ NK cells that distinguished stable from intermittent remission (Figure 1C), such that the proportion of CD8^+^CD57^+^ NK cells was significantly increased in stable RA remission (Figure 1D).

To perform a cross-platform validation of our mass cytometry signature, as well as to provide additional phenotypic granularity, we designed an extended NK cell phenotyping panel for spectral flow cytometry on a validation set of samples from the REMIRA cohort. In addition, we included patients with active disease and age-matched healthy controls to compare extreme phenotypes. A summary of the demographics, clinical information, and treatments for each group is provided in Supplemental Table 4.

It is interesting to note that, while tender joint count (TJC) and erythrocyte sedimentation rate (ESR) levels in active disease may be contributing to increased DAS28 scores, the largest difference across groups is reflected in patient reported outcomes. In general, the number of swollen joints and inflammatory markers remained low across groups, suggesting that clinical inflammatory markers were unable to capture the underlying biological heterogeneity driving different disease outcomes.

First, samples were enriched for NK cells by magnetic depletion of CD3^+^ and CD19^+^ lymphocytes and stained using a spectral flow panel as detailed in Supplemental Table 2. To validate the increased CD8^+^CD57^+^ subsets observed in remission, CD56^bright^ and CD56^dim^ NK cell subsets were first gated based on expression of CD56 and CD16 (Figure 1E), followed by analysis of the coexpression of CD8 and CD57, subdividing total CD56^dim^ NK cells into 4 discrete subsets. We observed significantly increased proportions of CD8^+^CD57^+^ NK cells in the blood of patients in stable remission when compared with intermittent remission and active disease (Figure 1F), validating the mass cytometry data. While proportions of CD8^+^CD57^+^ NK cells in healthy controls were more variable, median values aligned more to those observed in stable remission. To assess the stability of this NK cell subset in patients in stable remission over time, we analyzed the proportion of CD8^+^CD57^+^ NK cell subsets in paired samples taken at baseline and after 12 months (Figure 1G). No statistically significant differences were observed between time points, suggesting that peripheral blood CD8^+^CD57^+^ NK cells in sustained remission were stable over time.

We noted that the increased frequency of CD8^+^CD57^+^ cells in stable remission was associated with a decrease in the proportion of CD8^–^CD57^–^ NK cells, especially when compared with active disease (Supplemental Figure 1A). No differences in the proportion of CD8^+^CD57^–^ and CD8^–^CD57^+^ NK cells were observed across RA disease activity states, although — when compared with healthy controls — subsets were reduced and increased, respectively (Supplemental Figure 1, B and C). Lastly, we found no differences in the proportion of CD56^bright^ NK cells (Supplemental Figure 1D). These data suggest that CD8^+^CD57^+^ NK cells are expanded in the periphery of patients with RA in sustained remission and that this signature is stable over time.

### CD8^+^CD57^+^ NK cells expressing KIR2DL1 are expanded in stable remission.

NK cell surface receptors are commonly classified according to their associated signaling responses as either activating or inhibitory. The fundamental importance of inhibitory receptor signaling in establishing the functional capacity of NK cells, a process referred to as NK cell functional licencing, is underpinned by the hypofunctional state of NK cells lacking expression of inhibitory receptors (18).

**Figure 2:**
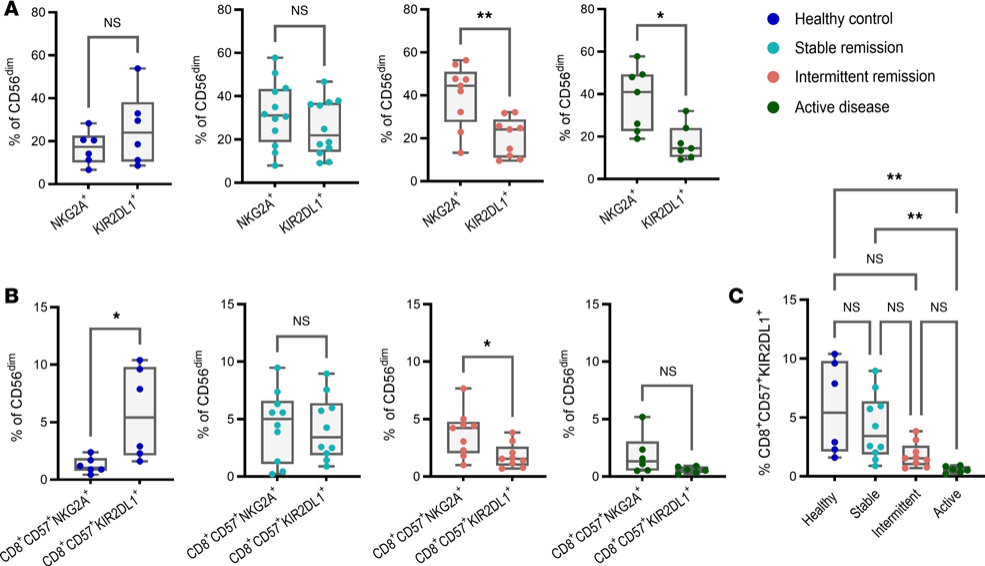
CD8^+^CD57^+^ NK cells expressing KIR2DL1 are expanded in stable remission. (**A**) The proportion of total CD56^dim^ NK cells that are NKG2A^+^ compared with KIR2DL1^+^. (**B**) The proportion of total CD56^dim^ NK cells that are CD8^+^CD57^+^NKG2A^+^ compared with CD8^+^CD57^+^KIR2DL1^+^. (**C**) CD8^+^CD57^+^KIR2DL1^+^ NK cells as a percentage of CD56^dim^. Whiskers on plots represent minimum to maximum values. *P* values were determined by using the Mann-Whitney *U* test (**A** and **B**) and Kruskal-Wallis test with Dunn’s multiple-test correction (**C**). *P < 0.05, **P < 0.01.

To investigate whether the composition of NK cells from stable remission was associated with a specific inhibitory receptor profile, we interrogated the spectral flow cytometry data derived from our validation sample set (Figure 1) to compare the proportion of total CD56^dim^ NK cells expressing NKG2A with those expressing KIR2DL1 (selected as a representative KIR based on evidence suggesting that it has the strongest licensing effect of all KIRs; refs. 19, 20) (Figure 2A). The results demonstrate that for stable remission, like healthy controls, there were equal proportions of CD56^dim^ NK cells expressing both inhibitory receptors, suggesting a more diverse inhibitory receptor profile. In contrast, intermittent remission, like active disease, demonstrated a redistribution in inhibitory receptor expression reflected in an increased proportion of CD56^dim^ NK cells preferentially expressing NKG2A.

To assess the contribution of the remission-associated CD8^+^CD57^+^ subsets to compositional changes in inhibitory receptor expression, we compared the proportion of these subsets expressing KIR2DL1 to those expressing NKG2A as a percentage of total CD56^dim^ NK cells (Figure 2B). NK cells from healthy controls were composed of greater proportions of CD8^+^CD57^+^ expressing KIR2DL1, consistent with the literature demonstrating an increase in KIR expression associated with CD57^+^ NK cell subsets (21). In contrast, both intermittent remission and active disease demonstrated increased proportions of CD8^+^CD57^+^ subsets expressing NKG2A, in keeping with the overall expansion of NKG2A^+^CD56^dim^ NK cells observed in these groups. Interestingly, NK cells from patients in stable remission were composed of equal proportions of CD8^+^CD57^+^ subsets expressing both inhibitory receptors, suggesting that remission is associated with a more heterogenous pool of this expanded subset. Nonetheless, the overall proportion of CD8^+^CD57^+^ NK cells expressing KIR2DL1 was increased in stable remission, particularly when compared with active disease (Figure 2C), allowing us to conclude that CD8^+^CD57^+^KIR2DL1^+^ NK cells in peripheral blood are associated with sustained RA remission.

### CD8^+^CD57^+^ NK cells display increased levels of CD226 expression consistent across RA disease activity states.

**Figure 3:**
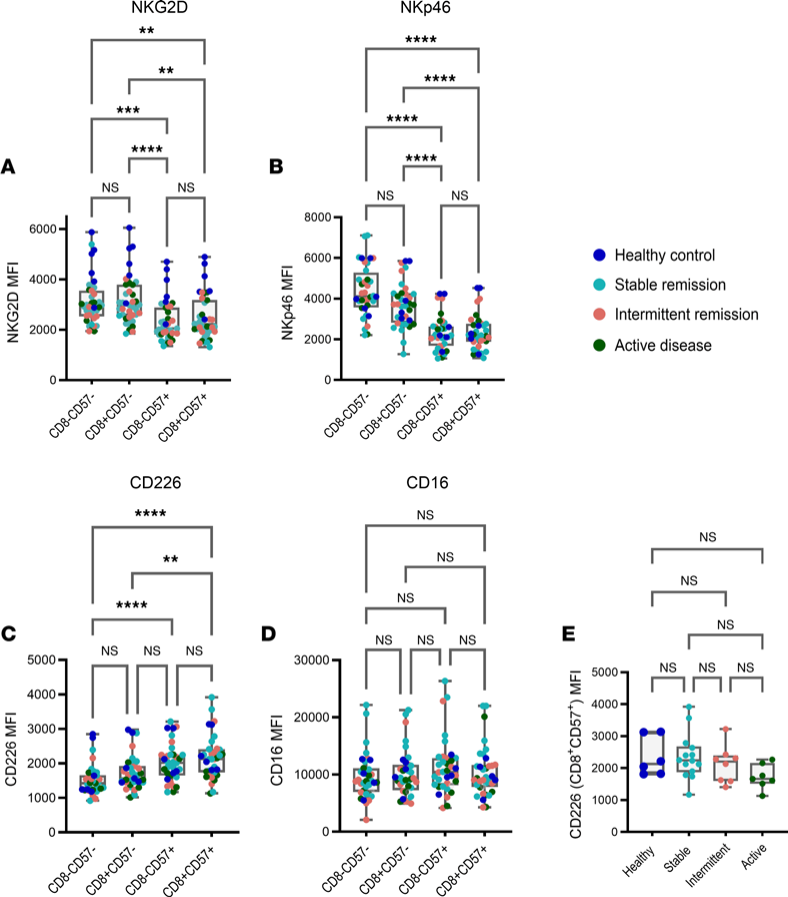
CD8^+^CD57^+^ NK cells display higher levels of CD226 expression consistent across RA disease activity states. (**A**–**D**) Median fluorescence intensity (MFI) of NKG2D, NKp46, CD226, and CD16 on NK cell subsets subdivided by CD8 and CD57 expression. (**E**) MFI of CD226 on CD8^+^CD57^+^ NK cells. Whiskers on plot represent minimum to maximum values. All *P* values were determined using the Kruskal-Wallis test with Dunn’s multiple-test correction. **P < 0.01, ***P < 0.001, ****P < 0.0001.

Next, we further interrogated the spectral flow cytometry data derived from our validation sample set to investigate whether CD8^+^CD57^+^ NK cells in stable remission were associated with changes in activating receptor expression. Analysis of the proportion of total CD56^dim^ NK cells expressing NKG2D, NKp46, CD16, and CD226 revealed that more than 95% of circulating NK cells express each given activating receptor consistent across disease activity states (Supplemental Figure 2A). Thus, we investigated whether the levels of receptor expression (as indicated by median fluorescence intensity [MFI]) differed across NK cell subsets subdivided by CD8 and CD57 expression. Our results reveal a differential MFI according to CD57 expression status, with levels of NKG2D and NKp46 significantly reduced within CD57^+^ NK cells while the expression of CD226 was increased (Figure 3, A–C). No differences in the level of CD16 expression was observed (Figure 3D). These differences were not associated with CD8 expression, implying that changes are associated exclusively with differentiation.

Although stable remission was associated with expansion in the overall proportions of CD8^+^CD57^+^ NK cells, CD226 MFI was consistent across the spectrum of RA disease activity (Figure 3E), suggesting that changes in functional capacity are controlled through compositional shifts in the proportion of subsets expressing CD226 rather than changes in the expression at a subset level. This was similar for expression levels of NKG2D, NKp46, and CD16 (Supplemental Figure 2B). In conclusion, CD8^+^CD57^+^ NK cells seen in stable remission were defined by lower levels of NKG2D and NKp46 and by higher levels of CD226 relative to their CD57^–^ counterparts.

### Degranulation with reduced proinflammatory cytokine expression is a functional characteristic of NK cells from stable remission.

Thus far, we have identified that the expansion of an NK cell subset in peripheral blood is associated with sustained RA remission. The question arises as to whether changes in NK cell subsets in the blood of patients in remission represent solely a biomarker of treatment response or whether there may also be differences in their functional capacity, suggesting a role for modulation of NK cell effector function in inducing disease remission.

**Figure 4:**
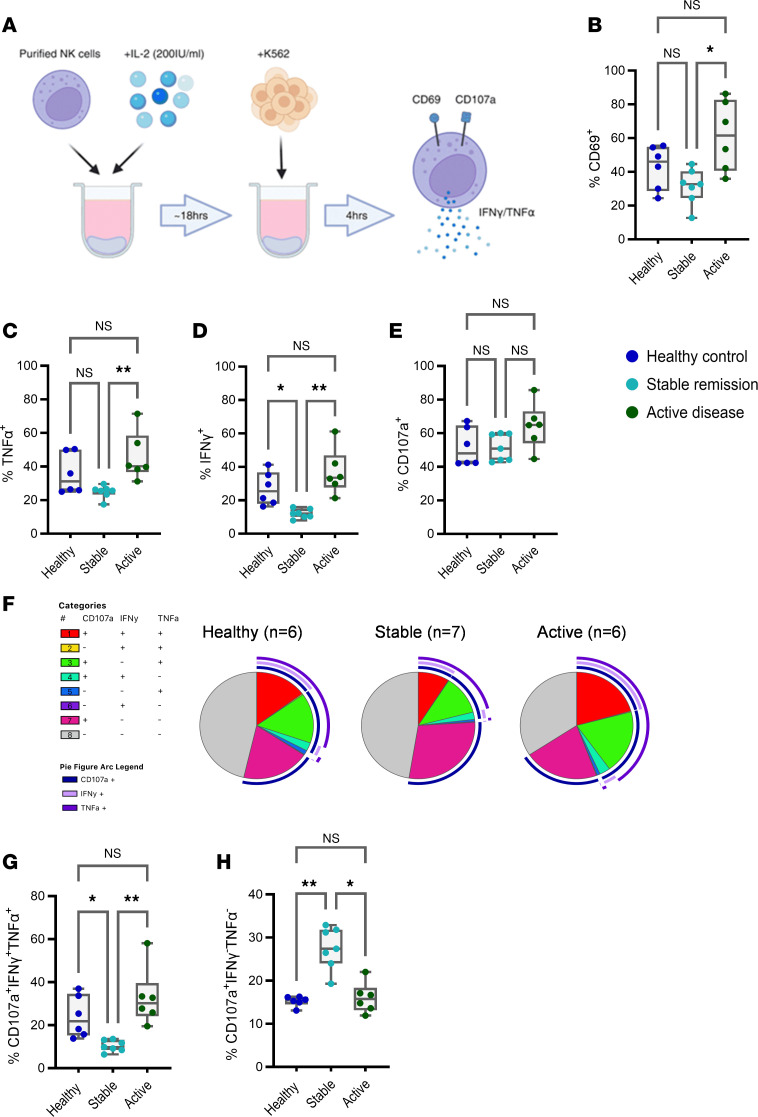
NK cell subsets with normal degranulation responses and reduced proinflammatory cytokine expression are elevated in stable remission. (**A**) NK cells from healthy donors (*n* = 6), stable remission (*n* = 7), and active disease (*n* = 6) were purified and stimulated with IL-2 (200 IU/mL) and incubated with K562 cells. (**B–E**) The proportions of NK cells expressing CD69, TNF-α, IFN-γ, and CD107a. (**F**) SPICE analysis defines 8 distinct functional subtypes based on TNF-α, IFN-γ and CD107a expression. (**G** and **H**) the proportion of CD107a^+^IFN-γ^+^TNF-α^+^ and CD107a^+^IFN-γ^–^TNF-α^–^ functional subsets. Whiskers on plots represent minimum to maximum values. All *P* values were determined using the Kruskal-Wallis test with Dunn’s multiple-test correction. *P < 0.05, **P < 0.01.

To investigate whether remission is associated with changes in NK cell functionality, we purified NK cells from patients in stable remission, stimulated them for 16 hours with IL-2 to enhance functional responses, and then cocultured them with K562 target cells prior to analysis by flow cytometry (Figure 4A). Phenotyping panel information is summarized in Supplemental Table 3. We also included data from healthy controls and active RA disease to compare extreme phenotypes (sample summary information is detailed in Supplemental Table 5). Cellular activation was evaluated by surface CD69 expression and proinflammatory cytokine expression (IFN-γ and TNF-α), while surface expression of CD107a (LAMP-1) was used as a readout for NK cell degranulation.

CD69 expression revealed that, in response to IL-2 stimulation and K562 target cell interaction, NK cells from patients with RA in stable remission exhibited levels of activation like healthy controls, while NK cells from patients with RA with active disease displayed elevated levels of activation (Figure 4B). A similar pattern of response was observed for expression of TNF-α (Figure 4C). Importantly, IFN-γ–expressing NK cells were significantly reduced in stable remission when compared with healthy donors and were even lower than those observed in NK cells from patients with active disease (Figure 4D), while surface expression of CD107a did not show any statistically significant differences between groups (Figure 4E). We conclude that NK cells from patients in stable remission retain the capacity for degranulation in response to target cell engagement but produce much lower levels of inflammatory cytokines. In addition, functional responses of CD56^bright^ NK cells revealed no differences in cytokine expression and degranulation among groups (Supplemental Figure 3, A–C), suggesting that reductions in IFN-γ expression in stable remission was specific to the CD56^dim^ NK cell compartment.

To evaluate the coexpression patterns of both proinflammatory cytokines and degranulation, Simplified Presentation of Incredibly Complex Evaluations (SPICE) analysis was performed (22). Boolean gating of NK cell proportions based on expression of CD107a, IFN-γ, and TNF-α gave rise to 8 distinct functional NK cell subtypes (Figure 4F and Supplemental Figure 4). In response to K562 target cell interaction, NK cells from stable remission exhibited reduced proportions of polyfunctional CD107a^+^IFN-γ^+^TNF-α^+^ subsets (Figure 4G), while proportions of CD107a^+^IFN-γ^–^TNF-α^–^ subsets where significantly increased (Figure 4H), indicating that NK cells from patients with RA in remission gained a specific functional subset with an intact ability to degranulate in the absence of proinflammatory cytokine expression.

Finally, given that coexpression of CD57 and CD8 defined a subset of NK cells that were expanded in the blood of patients with RA in stable remission, we analyzed whether the proportion of CD107a^+^IFN-γ^+^TNF-α^+^ polyfunctional subsets was different between CD8^–^ and CD8^+^ subsets (Supplemental Figure 5A) and CD57^–^ and CD57^+^ subsets (Supplemental Figure 5B). Consistent across all groups, the expression of CD8 was associated with increased polyfunctional NK cells, indicating that CD8^+^ NK cells exhibit enhanced effector functions when compared with CD8^–^ subsets. No significant differences in polyfunctional NK cells were observed between CD57^–^ and CD57^+^ NK cells.

### A distinct phenotypic subset associated with in vitro polyfunctional responses is reduced in stable remission.

Given the fundamental link between NK cell subset diversity and functional responses (23), we further interrogated our functional dataset (as detailed in Figure 4) to investigate whether CD107a^+^IFN-γ^+^TNF-α^+^ polyfunctional NK cells (Figure 4G) were associated with a specific in vitro induced phenotypic subset.

**Figure 5:**
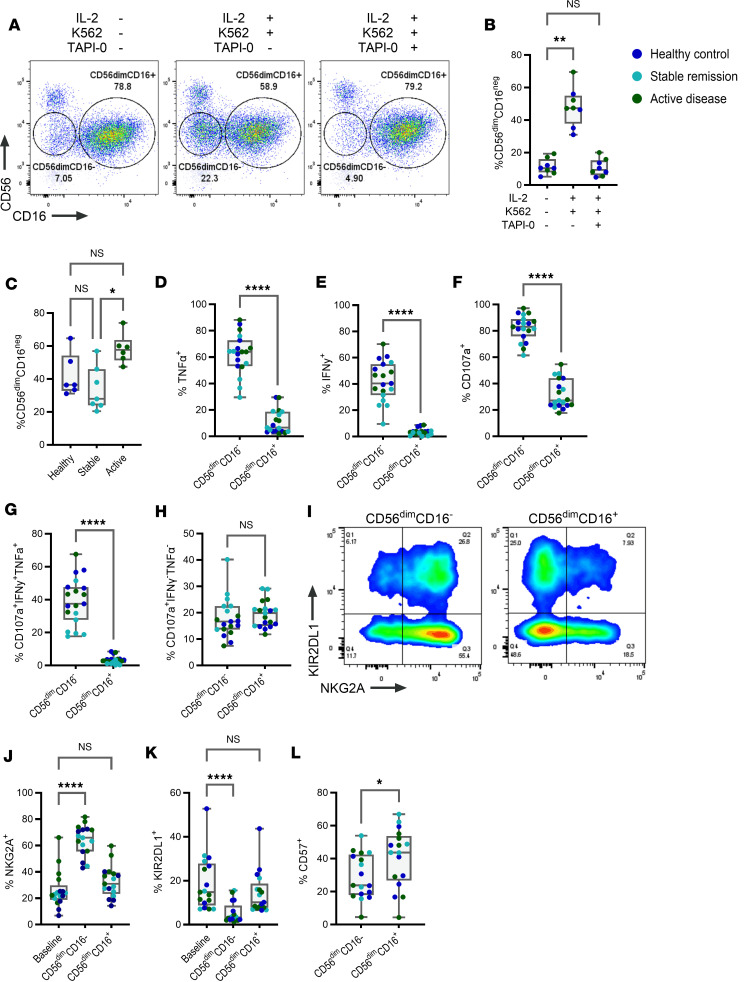
A distinct phenotypic subset of NK cells associated with in vitro polyfunctional responses is reduced in stable remission. (**A** and **B**) The in vitro induction of a CD56^dim^CD16^–^ NK cell population, which arises in response to IL-2 stimulation and K562 interaction, is reduced upon treatment with TAPI-0 (5 μM). (**C**) The proportion of CD56^dim^CD16^–^ NK cells in response to IL-2 stimulation and K562 interaction. (**D**–**H**) Comparison of the proportion of NK cells expressing TNF-α, IFN-γ, and CD107a (**D**–**F**) as well as CD107a^+^IFN-γ^+^TNF-α^+^ (**G**) and CD107a^+^IFN-γ^–^TNF-α^–^ (**H**) functional subsets between CD56^dim^CD16^–^ and CD56^dim^CD16^+^ NK cells. (**I**) Representative flow plot demonstrating the expression of KIR2DL1 and NKG2A. (**J** and **K**) Differences in the expression from baseline (IL-2^–^K562^–^) of NKG2A, and KIR2DL1 between CD56^dim^CD16^–^ and CD56^dim^CD16^+^ subsets. (**L**) Comparison of the proportion of CD57^+^ NK cells between CD56^dim^CD16^–^ and CD56^dim^CD16^+^ subsets. Whiskers on plots represent minimum to maximum values. All *P* values were determined using the Kruskal-Wallis test with Dunn’s multiple-test correction. *P < 0.05, **P < 0.01, ****P < 0.0001

Analysis revealed that, in response to IL-2 stimulation and K562 target cell interaction, a CD56^dim^CD16^–^ NK cell subset emerged (Figure 5A). Evidence has shown that, in response to activation, surface expression of CD16 on NK cells is reduced, driven by enzymatic cleavage via a Disintegrin and Metalloprotease 17 (ADAM17) (24). We hypothesized that the in vitro emergence of this CD56^dim^CD16^–^ subset would occur because of ADAM17-mediated CD16 receptor shedding. To test this, IL-2–stimulated NK cells were cocultured with K562 cells in the presence of TAPI-0, a known inhibitor of ADAM17 function (Figure 5A). In the presence of an inhibitor, the proportion of CD56^dim^CD16^–^ NK cells was significantly reduced, indicating that TAPI-0 inhibited surface CD16 receptor loss (Figure 5, A and B). Furthermore, NK cells from stable remission demonstrated significant reductions in the frequency of CD56^dim^CD16^–^ NK cell subsets when compared with active RA (Figure 5C), despite no differences in the baseline expression of CD16 (Supplemental Figure 6).

Next, we analyzed the differences in functional responses of CD56^dim^CD16^–^ and CD56^dim^CD16^+^ NK cells to determine if either subset was associated with polyfunctionality. Increased expression of TNF-α, IFN-γ, and CD107a was observed in CD56^dim^CD16^–^ NK cells (Figure 5, D–F), resulting in a corresponding increase in CD107a^+^IFN-γ^+^TNF-α^+^ subsets exclusively in CD56^dim^CD16^–^ NK cells (Figure 5G). No significant differences were observed in the proportion of CD107a^+^IFN-γ^–^TNF-α^–^ subsets between CD56^dim^CD16^–^ and CD56^dim^CD16^+^ NK cells (Figure 5H). Thus, we can confirm that, in response to IL-2 stimulation and K562 target cell interaction, ADAM17-mediated shedding of CD16 gives rise to a CD56^dim^CD16^–^ NK cell subset that is associated with polyfunctional NK cell responses and that this subset is reduced in patients with RA in sustained remission.

Given the importance of inhibitory receptor expression in controlling NK cell effector functions, we next investigated whether CD56^dim^CD16^–^ and CD56^dim^CD16^+^ NK cells were enriched for subsets expressing specific inhibitory receptors and whether this expression differed when compared with baseline. Significant increases in the proportion of NKG2A^+^ NK cells with a corresponding reduction in the proportion of KIR2DL1^+^ NK cells were observed within CD56^dim^CD16^–^ NK cells when compared with baseline expression, while no differences in inhibitory receptor expression was observed within CD56^dim^CD16^+^ NK cells (Figure 5, I–K). In addition, we observed a decreased proportion of CD57^+^ NK cells within the CD56^dim^CD16^–^ subset when compared with CD56^dim^CD16^+^ cells (Figure 5L). These data indicate that CD56^dim^CD16^–^ NK cells were composed of a predominant NKG2A^+^KIR^–^CD57^–^ phenotype, while CD56^dim^CD16^+^ NK cells have a more diverse repertoire of inhibitory receptor expression, which aligned with that of baseline, unstimulated NK cells.

Thus, in response to IL-2 stimulation and K562 target cell interaction, ADAM17-mediated shedding of CD16 gave rise to a CD56^dim^CD16^–^ NK cell subset with a predominant NKG2A^+^KIR^–^CD57^–^ phenotype, associated with polyfunctional effector function and inflammatory responses. The corresponding CD56^dim^CD16^+^ NK cell subset phenotypically aligned better with baseline unstimulated NK cells while still exhibiting the functional capacity for degranulation in the absence of proinflammatory cytokine expression. Furthermore, we demonstrate that such subsets were unevenly represented across RA disease activity states, with active disease characterized by a higher proportion of CD56^dim^CD16^–^ NK cells, while NK cells from stable remission were composed of higher proportions of CD56^dim^CD16^+^ NK cells.

### NK cell subsets associated with stable remission are reduced in inflamed RA joints.

**Figure 6:**
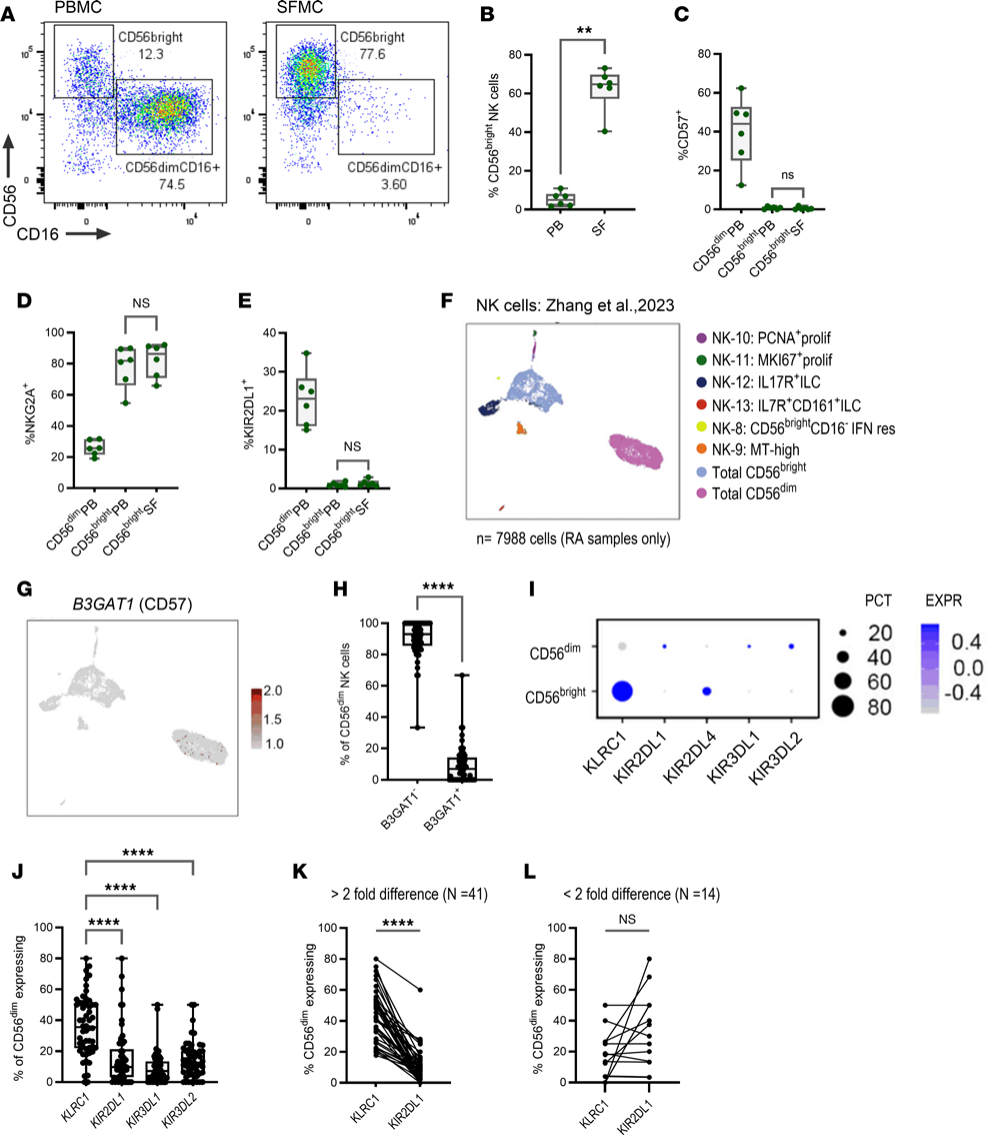
Phenotypic features associated with sustained remission are reduced on NK cells from the joint of active RA. (**A**) Expression of CD56 and CD16 defines CD56^bright^CD16^–^ and CD56^dim^CD16^+^ subsets between paired peripheral blood (PB) and synovial fluid (SF) (*n* = 6). (**B**) The proportion of CD56^bright^ NK cells between PB and SF. (**C**–**E**) The expression of CD57, NKG2A, and KIR2DL1 across CD56^dim^ and CD56^bright^ NK cells in PB and CD56^bright^ NK cells in SF. (**F**) UMAP plot demonstrating 8 transcriptional NK Cell/ILC clusters derived from single-cell RNA-Seq on synovial tissue from patients with active RA. (**G**) UMAP feature plot showing the expression of *B3GAT1* across CD56^bright^ and CD56^dim^ clusters. (**H**) Comparison of the proportion of CD56^dim^ NK cells not expressing and expressing *B3GAT1* across individual tissue samples (*n* = 63). (**I**) Bubble plots demonstrating the percentage of cells expressing genes for specific inhibitory receptors and their expression levels across CD56^bright^ and CD56^dim^ clusters. (**J**) Comparing the proportion of CD56^dim^ NK cells expressing *KLRC1* to cells expressing genes encoding each corresponding KIR. (**K** and **L**) The proportion of NK cells expressing KLRC1 versus KIR2DL1 at a greater than or less than 2-fold difference. Whiskers on plots represent minimum to maximum values. All *P* values were determined using the Mann-Whitney *U* test (**B**–**E** and **H**), the Kruskal-Wallis test with Dunn’s multiple-test correction (**J**), and the Wilcoxon matched-pairs signed-rank test (**K** and **L**). **P < 0.01, ****P < 0.0001.

To investigate whether NK cell phenotypes associated with remission are found within inflamed synovial joints, we profiled NK cells from paired peripheral blood (PB) and synovial fluid (SF) of patients with RA with active disease (sample summary information is provided in Supplemental Table 6). The expression of CD56 and CD16 revealed striking differences in the composition of NK cell subsets (Figure 6A) in which SF was enriched in CD56^bright^CD16^–^ NK cells (Figure 6B) while PB was enriched in CD56^dim^CD16^+^ NK cells (Supplemental Figure 7A). To investigate whether the difference in composition of CD16^–^ NK cells between PB and SF could in part be explained by changes in the levels of ADAM17 (given its role in CD16 receptor shedding), we measured the levels of soluble ADAM17 by ELISA in paired serum and SF samples from patients with active RA. Results demonstrate that, while we were able to detect appreciable levels of ADAM17, there were no differences observed between serum and fluid samples (Supplemental Figure 7B). Thus, the predominant NK cell subset observed in SF is likely a canonical CD56^bright^ subset (which is inherently CD16^–^) rather than a subset that has undergone CD16 loss. This is further supported by an overlap in the phenotype of CD56^bright^ NK cells in the fluid with that of those in the blood in terms of CD57, NKG2A, and KIR2DL1 expression (Figure 6, C–E). These data suggest that CD8^+^CD57^+^KIR2DL1^+^CD56^dim^ NK cell subsets, associated with stable remission, are absent from SF of patients with active RA.

To corroborate this observation, we interrogated NK cell signatures from a single-cell RNA-Seq dataset derived from synovial tissue biopsies of patients with active RA (25). From the 14 NK cell transcriptional clusters described, we merged clusters corresponding to CD56^dim^ and CD56^bright^ NK cells to generate 8 clusters comprising discrete populations that aligned with our cytometric data (Figure 6F).

Unlike SF, CD56^dim^ NK cells reside in high numbers within synovial tissue, with equal proportions of cells represented in both CD56^dim^ and CD56^bright^ transcriptional clusters (Supplemental Figure 7C). However, analysis of *B3GAT1*, the gene encoding CD57, revealed minimal expression within CD56^dim^ NK cell clusters (Figure 6, G and H). Thus, although CD56^dim^ NK cells were found within high numbers in synovial tissue, they were predominantly CD57^–^ at a transcriptional level.

Next, we interrogated the expression of genes encoding NKG2A (*KLRC1*), KIR2DL1 (*KIR2DL1*), KIR2DL4 (*KIR2DL4*), KIR3DL1 (*KIR3DL1*), and KIR3DL2 (*KIR3DL2*) across CD56^bright^ and CD56^dim^ NK cell clusters (Figure 6I). CD56^bright^ NK cell clusters predominantly expressed *KLRC1* in the absence of genes encoding all KIRs (Figure 6I and Supplemental Figure 7D), although there was a select number of patients expressing *KIR2DL4*, the gene encoding a major activating KIR (Supplemental Figure 7E). While a small proportion of CD56^dim^ NK cells demonstrated expression of genes encoding *KIR2DL1*, *KIR3DL1*, and *KIR3DL2*, a larger proportion of cells expressed *KLRC1* (Figure 6J), suggesting that the predominant phenotype of CD56^dim^ NK cells within synovial tissue was NKG2A^+^KIR^–^ at a transcriptional level. Additionally, given our observations regarding preferential expression of KIR2DL1 at the protein level in stable remission, we separated samples into those showing greater than or less than 2-fold difference in the number of cells expressing *KLRC1* versus *KIR2DL1* (Figure 6, K and L). We observed that approximately 75% of synovial tissue samples had higher proportions of CD56^dim^ NK cells expressing *KLRC1*.

Thus, while high numbers of CD56^dim^ NK cells were found within inflamed synovial tissue, their phenotype appears distinct from that in blood, with a predominant NKG2A^+^CD57^–^KIR^–^ phenotype. These features align with our in vitro phenotype associated with polyfunctional NK cell effector responses (Figure 5). We can conclude that NK cell phenotypes associated with RA remission in the blood are reduced from the site of active inflammation in RA.

## Discussion

Variable outcomes associated with drug tapering and withdrawal in patients with RA highlight the need for a more precision-medicine based approach. Here, we performed unbiased, high-dimensional immune phenotyping and identified CD8^+^CD57^+^CD56^dim^ NK cells as a potentially novel and distinctive PB signature associated with stable remission. NK cells are innate immune effectors belonging to a class of immune cell subsets collectively referred to as innate lymphoid cells (ILCs) (26). ILC subsets are immune cells of lymphoid origin but are distinct in that they lack expression of somatically rearranged T or B cell antigen receptors. ILC subsets are commonly subdivided into three groups (Group 1–3 ILCs) based on distinct functional properties of each group (27). NK cells alongside type 1 ILC subsets (ILC1s) constitute the major members of Group 1 ILCs defined based on their shared requirement of T-bet expression to promote their development as well as functional overlap in the expression of cytotoxic mediators and IFN-γ (27).

Similar NK cell phenotypes associated with favorable disease outcomes have been reported in the literature, such as CD8^+^CD57^+^ NK cells associated with slower HIV-1 disease progression (28), a CD8^+^ NK cell transcriptomic signature associated with periods of remission in relapsing remitting multiple sclerosis (29), and CD8^+^ NK cells associated with response to anti–TNF-α therapy in ankylosing spondylitis (30). Thus, it is likely that NK cells may represent a conserved signature of disease remission across multiple disease settings.

We have identified that blood-derived CD56^dim^ NK cells in stable remission exhibited increased expansions of CD8^+^CD57^+^ NK cells expressing KIR2DL1. The fundamental importance of inhibitory receptor expression for inducing NK cell effector functionality is reflected in the hyporesponsive state of NK cells lacking either NKG2A or KIR (18). Differences in both the type and level of expression of KIRs, underpinned by genetic diversity, has been implicated in disease processes, likely due to variability in different KIRs to promote the functional capacity of NK cell subsets (31). Whether expression of KIR2DL1 contributes to the emergence of a functional subset capable of maintaining disease remission in RA remains to be addressed. Furthermore, we also observed that CD56^dim^ NK cells from healthy controls exhibited expansions of CD8^+^CD57^+^ NK cells preferentially expressing KIR2DL1 while CD8^–^CD57^–^ NK cells preferentially expressed NKG2A (data not shown). This is in keeping with the literature that demonstrates that changes in the expression of inhibitory receptors are linked to NK cell differentiation (17), such that CD57^–^ NK cells have higher NKG2A expression while CD57^+^ NK cells express higher levels of KIR. The increased NKG2A expressing CD57^+^ NK cells in intermittent remission and active disease suggests that, in the context of KIR2DL1 expression, this association was lost. However, it is worth noting that, due to the limited coverage of KIRs used in this study, increased proportions of NKG2A-expressing CD8^+^CD57^+^ NK cells associated with reduced KIR2DL1^+^ subsets may reflect the presence of alternatively expressed KIRs. Additional extended KIR phenotyping would enable an investigation of whether alternative KIRs are present within more active clinical states.

In vitro functional assessment revealed that, in response to IL-2 stimulation and K562 target cell interaction, CD56^dim^ NK cells from patients with RA in stable remission exhibit a reduction in inflammatory cytokine expression and reduced proportions of polyfunctional NK cell responses. We have also demonstrated that ADAM17-mediated shedding of CD16 gave rise to a CD56^dim^CD16^–^ subset associated with polyfunctional responses, which was reduced from individuals in stable remission. ADAM17-mediated modulation of CD16 receptor expression has been described as a key NK cell immune checkpoint, acting as a negative regulator of the effector functions of activated NK cells (32). Indeed, inhibition of ADAM17 has been shown to result in enhanced NK cell functional responses (33). Therefore, CD16 surface shedding corroborates the increased activation state of polyfunctional NK cells observed in active RA, while the reduced levels of shedding observed in remission may suggest a more regulated effector response. Increased proportions of CD107a^+^TNF-α^–^IFN-γ^–^ subsets were associated with sustained remission, suggesting a predominant functional subtype characterized by degranulation in the absence of proinflammatory cytokine expression. Such functionality, which we have coined “silent degranulation,” could be considered advantageous in the context of disease remission by maintaining NK cell degranulation responses, thereby contributing to homeostatic immune surveillance, while restraining the proinflammatory burden. Identifying the molecular mechanism controlling cytokine suppression in NK cells from stable remission is an area of further research with the potential for high translational effects, especially if this turns out to be a fundamental control mechanism common to multiple disease entities.

Given the increased proportion of CD8^+^CD57^+^ NK cells in stable remission, a key question arises as to what extrinsic factors could contribute to the emergence of this subset. While regulation of CD8^+^ NK cell populations remain poorly understood, evidence supports chronic viral infection, particularly human cytomegalovirus (HCMV) infection, as one of the best recognized clinical scenarios associated with expansion of CD57^+^ NK cell subsets (34). Indeed, the host response to HCMV infection has been associated with the expansion of a specialized NK cell subset with adaptive features defined phenotypically by elevated levels of CD57, as well as the expression of the activating receptor NKG2C, although subsets with similar adaptive-like features not expressing NKG2C have been reported (35). Thus, one could hypothesize that CD8^+^CD57^+^ NK cells in stable remission represent a key immune cell subset with intact homeostatic defence systems to control latent viral infection, whose reactivation is associated with disease flares. This raises the intriguing possibility that CD8^+^CD57^+^ NK cells could be more than just a signature of stable remission but could be actively contributing to a sustained remission state.

The role of NK cell cytotoxicity in mediating regulation of adaptive immune responses, in particular modulation of CD4^+^ and CD8^+^ T cell responses, could also represent a mechanism whereby NK cells could contribute to immune homeostasis (36). Defining whether NK cells in stable remission with the capacity for “silent degranulation” could contribute to controlling expansion of autoreactive T cells without the release of proinflammatory cytokines would be worthy of further investigation. Alternatively, NK cells have been found to reside in high numbers within lymph nodes whereby they modulate dendritic cell function (37), indicating that remission-associated NK cell subsets could regulate autoimmunity by influencing autoantigen presentation in secondary lymphoid organs. Direct crosstalk between NK cells and fibroblast-like synoviocytes has also been documented (38), indicating a potential role for NK cell–mediated clearance of these pathogenic stromal cells.

Whether NK cell regulation would occur in the inflamed joint or alter the immune response at an alternative anatomical location would also require addressing. We have shown that NK cells within the joint of active RA are phenotypically distinct (at both the protein and transcriptional level) from our CD8^+^CD57^+^KIR2DL1^+^ remission signature, indicating the absence of potential regulatory components. Given that the expression of CD57 is associated with a reduced proliferative rate and decreased responsiveness to cytokine stimulation (39), we hypothesize that the reduced presence of CD57^+^ NK cells in the RA joint might be explained by their failure to differentiate in an inflamed microenvironment. In this inflammatory setting, chronic cytokine stimulation would favor the accumulation of NKG2A^+^KIR^–^CD57^–^ NK cell phenotypes owing to the increased proliferative rate and enhanced metabolic fitness associated with NKG2A expression (40). This provides further incentive to define pathways for therapeutic control of inflammation within the RA joint that may permit CD8^+^CD57^+^ NK cells to differentiate and induce disease remission.

Lastly, given that remission in this study is maintained by treatment, there remains a need to examine the dynamics of these subsets in the context of treatment tapering/withdrawal to investigate whether these NK cell subsets are maintained in drug-free remission.

## Methods

### Sex as a biological variable.

Human samples were included from both female and male patients. A higher proportion of samples from female patients were included in each group to reflect the increased prevalence of RA observed in females.

### Study samples.

All samples for mass cytometry analysis (Figure 1, A–D), stable remission, intermittent remission, and active disease samples for spectral flow cytometry (Figure 1, E–G; Figure 2, and Figure 3) and stable remission samples from functional studies (Figure 4 and Figure 5) were derived from the REMIRA cohort. Participants with RA were recruited to the REMIRA study if they were diagnosed according to the 1987 revised American College of Rheumatology criteria (41). Specific inclusion criteria included: Disease duration of < 10 years, stable DMARD treatment for > 6 months and a DAS28 score of < 3.2 for a least 1 month before recruitment.

Active disease samples from functional studies (Figure 4 and Figure 5), paired PB and SF samples (Figure 6), and all healthy controls were derived from the METAbolic Control of the Immune Response (METACIR) study (REQ no. 18/LO/0399). Patients with RA (>18 years old) were recruited to the METACIR study after fulfilling the 2010 ACR/EULAR classification criteria for diagnosis of RA.

### Cell isolation.

PB mononuclear cells (PBMCs) and SF mononuclear cells (SFMCs) were isolated by density gradient centrifugation using Lymphoprep (Axis-Shield) (800*g* for 15 minutes). PBMC from the REMIRA study were cryopreserved in freezing medium containing 10% DMSO (MilliporeSigma) in 90% human AB serum (PAA Laboratories), while PBMC and SFMC from the METACIR study were cryopreserved in freezing medium containing 10% DMSO (MilliporeSigma) in 90%FBS (MilliporeSigma). All samples were stored in liquid nitrogen. Cell viability was routinely tested by Trypan blue exclusion, with a viability of > 85% considered acceptable.

### Mass cytometry staining and acquisition.

After thawing, dead cells were removed from samples using a dead cell removal kit (Miltenyi Biotec) according to manufacturer’s instructions. For subsequent wash steps, unfixed cells were centrifuged at 300*g* for 5 minutes and fixed cells at 800*g* for 5 minutes at room temperature (RT). In total, 3 × 10^6^ cells were stained with cisplatin (for live/dead discrimination) (StandardBioTools) in plain RPMI-1640 (Thermo Fisher Scientific) for 5 mins at RT and washed using RPMI-1640 (Thermo Fisher Scientific) supplemented with 10% FBS (MilliporeSigma) to quench. Cells resuspended in maxpar staining buffer (MSB) (StandardBioTools) were incubated with human FC block (BioLegend) for 10 minutes at RT followed immediately by addition of surface stain antibodies (Supplemental Table 1), incubated for 60 minutes at RT. Cells were then fixed and barcoded using the Cell-ID 20-Plex Pd barcoding kit (StandardBioTools) according to manufacture instructions. Barcoded cells from multiple samples were pooled and incubated overnight with Cell-ID Intercalator-Ir (Fluidigm) prepared at a 1:500 dilution using 2% PFA. Cells were washed twice with PBS and resuspend in 1 mL of freezing medium (10% FCS/DMSO) and stored at –80°C until acquisition.

For acquisition, samples were washed with PBS and resuspended in Maxpar CAS buffer (Fluidigm) with EQ Element Calibration Beads (Fluidigm) and acquired on a Helios mass cytometer (Fluidigm).

### Flow cytometric analysis.

For wash steps, unfixed cells were centrifuged at 300*g* for 5 minutes and fixed cells at 800*g* for 5 minutes at 4°C unless otherwise stated. Cells were stained with Fixable Viability Dye eFluor 455UV (eBioscience) for 15 minutes at 4°C, followed by surface staining for 20 minutes at 4°C. For intracellular staining, cells were fixed using the Foxp3/Transcription factor staining buffer set (eBioscience) according to the manufacture instructions and incubated with intracellular antibodies for 30 minutes at 4°C. Cells were washed and resuspended in FACS buffer for acquisition. FCS files were acquired on a Cytek Aurora spectral flow cytometer and analyzed using FlowJo (Tree Star Inc.).

Where indicated, PBMC samples were enriched for NK cells by depletion of CD3^+^ and CD19^+^ lymphocytes prior to staining. Cells were incubated with 0.25 μg of both biotin anti–human CD3 (BioLegend, 300403, UCHT1) and biotin anti–human CD19 (BioLegend, 302203, HIB19) followed by magnetic depletion using Dynabeads Biotin Binder (Thermo Fisher Scientific).

### NK cell degranulation assays.

NK cells were purified from PBMCs by negative isolation using an NK cell isolation kit (Miltenyi Biotec). The quality of purification was accessed by measuring the proportion of NK cells after magnetic isolation by flow cytometry. All samples displayed a purity of > 95%. In total, 1 × 10^5^ purified cells were cultured in complete media (RPMI-1640 [Thermo Fisher Scientific] supplemented with 10% FBS, 1× glutaMAX [Thermo Fisher Scientific] and 1% penicillin/streptomycin) only or complete media in the presence of 200 U mL^−1^ of recombinant human IL-2 (Proleukin, Novartis) for ~18 hours at 37°C and 5% CO_2_. After incubation, cells were equally split into 2 wells (~5 × 10^4^ per well) to perform stimulations in duplicate. For target cell stimulation, 2 × 10^4^ K562 cells (provided by S. Papa, King’s College London) were added (E:T ratio of 2.5:1). Brefeldin A and Monensin (both BioLegend) were added (1:1,000 dilution) in addition to anti–human CD107a (1:200 dilution; BioLegend, 328617, clone H4A3) and cells were incubated for 4 hours at 37°C and 5% CO_2_. Where indicated, 5 μm TAPI-0 (provided by A. Ivetic, King’s College London) was added to each well and incubated for 4 hours at 37°C and 5% CO_2_. Following incubation, cells from duplicate wells were combined and harvested into 5 mL FACS tubes for flow cytometry.

### Mass cytometry data analyses.

EQ element calibration beads were used for signal intensity normalization as previously described (42). Barcode signals were deconvoluted using the Fluidigm CyTOF software generating individual sample FCS files. Data preprocessing was performed using manual gating in FlowJo (Tree Star Inc.) as previously described (43). Data quality was assessed by inspecting for technical outliers using hilbertSimilarity (44), where similarity between samples is estimated based on composition. No outlier batches were detected using the outliers.ranking method (45) with a threshold value of 0.7. Quality checked cells were clustered using FlowSOM into 169 clusters, which were manually annotated into metaclusters by visual inspection of marker expression profiles. The Euclidian distance between clusters was calculated and used to construct a minimal spanning tree graph for visualization of generated clusters. Differential cluster abundance was performed using a generalized linear mixed model based on *N ~ Condition + Gender +offset(Log[Total])*, where *N* refers to the number of cells in the cluster and *Condition* refers to what clinical state cells were derived from. The term *offset(Log[Total])* was used to account for differences in the number of cells across samples. Lastly, to identify compositional shifts within clusters between clinical states and identify surface markers that drive these differences, we utilized the FreeViz method implemented in the RadViz R package as previously reported (46).

### Statistics.

The graphical abstract was created with Biorender.com. For flow cytometry, statistical analysis was performed using GraphPad prism. Mann-Whitney *U* tests were performed on 2-way comparisons. For multiple groups, the Kruskal-Wallis test was performed with Dunn’s multiple-comparison test applied for adjusted *P* values. Paired data were analyzed with the Wilcoxon matched-pairs signed-rank test. A significance threshold of *P* < 0.05 was applied throughout the manuscript.

### Study approval.

The REMIRA study was approved by the Wandsworth Research Ethics committee (REC:09/H0803/154), and the METACIR study was approved by the London Bloomsbury research committee (REC:18/LO/0399). Both studies were conducted according to the guidelines of the Declaration of Helsinki with written informed consent obtained for all patients.

### Data availability.

All raw data and associated code are available from corresponding authors on request. As per the original publication (25), CITE-Seq single-cell expression matrices and sequencing are available on Synapse (https://doi.org/10.7303/syn52297840). Values for data points shown in figures are reported in the Supporting Data Values file.

## Author contributions

CC designed, performed, and analyzed the experiments and prepared the manuscript. MM and APC conceived and ran the REMIRA study. YA, CBM, and APC provided bioinformatic support and contributed to experimental design and preparation of the manuscript. CS provided mass cytometry acquisition support and contributed to experimental design and manuscript preparation. KS and NG contributed to experimental design and preparation of manuscript. SR, AC, RB, and EP conceived the project, designed experiments, supervized research, and contributed to the manuscript preparation.

## Supplementary Material

Supplemental data

Supporting data values

## Figures and Tables

**Table 1:**
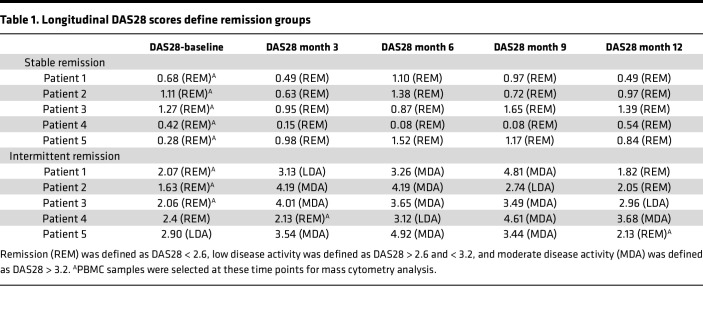
Longitudinal DAS28 scores define remission groups

**Table 2:**
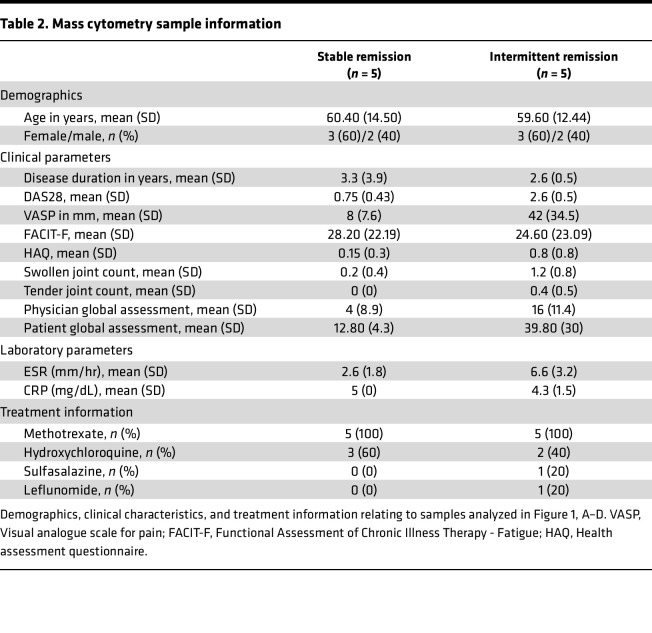
Mass cytometry sample information

## Version 1 10/17/2024

In-Press Preview

## Version 2 12/06/2024

Electronic publication
