# Supplementary material for: NK cell subsets define sustained remission in rheumatoid arthritis
Source: JCI Insight. 2024 Dec 6;9(23):e182390. doi: 10.1172/jci.insight.182390 (PMC11623943; doi:10.1172/jci.insight.182390)
Supplement: Supplemental data [file jciinsight-9-182390-s070.pdf]

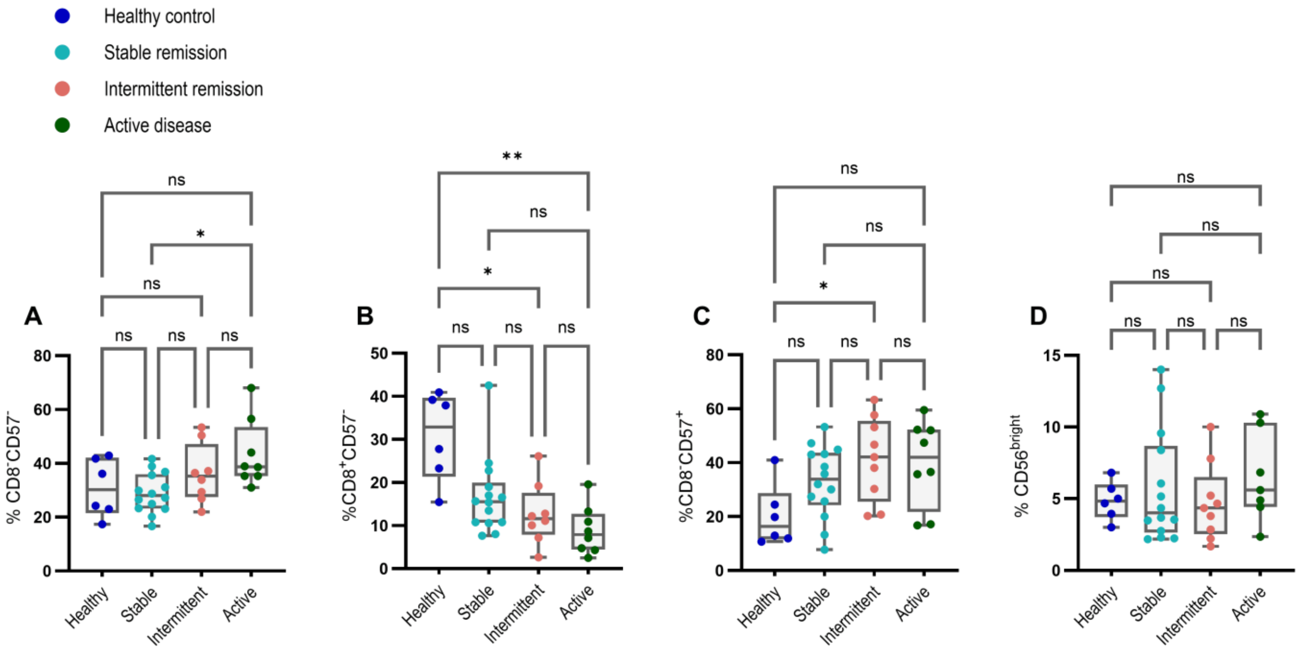

Supplementary Figure 1. Changes in CD56^{dim} NK cell subsets subdivided by CD8 and CD57 expression. The proportion of **(A)** CD8$^-$CD57$^-$, **(B)** CD8$^-$CD57$^+$, and **(C)** CD8$^+$CD57$^-$ NK cells. **(D)** The proportion of CD56^{bright} NK cells. Whiskers on plots represent min to max values. P values were determined by using the Kruskal-Wallis test with Dunns multiple test correction. *P <0.05, **P <0.01.

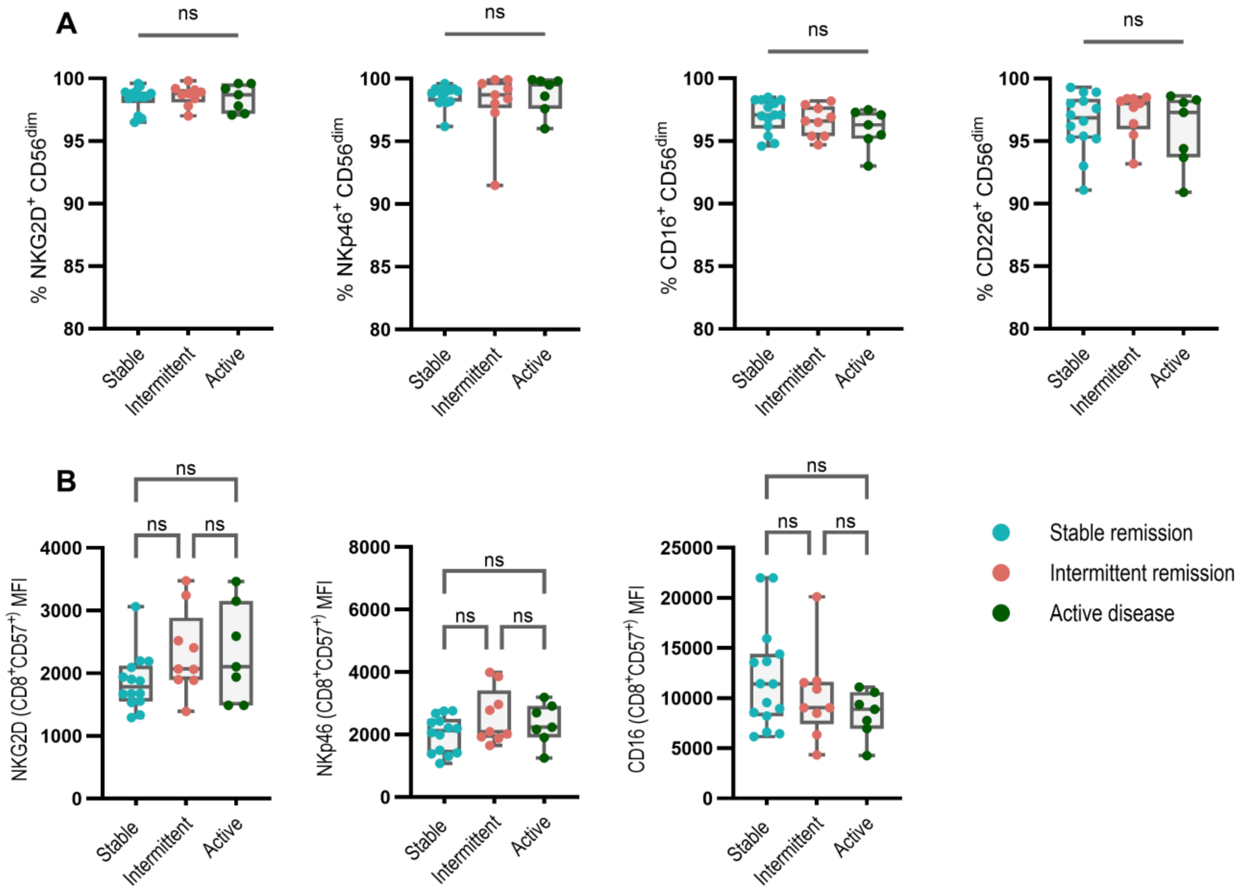

Supplementary Figure 2. NK cell activating receptor expression is consistent across disease activity states. (A) The proportion of total CD56^{dim} NK cells expressing NKG2D, NKp46, CD16 and CD226 **(B),** The MFI NKG2D, NKp46 and CD16 on CD8^{+}CD57^{+} NK cells. Whiskers on plots represent min to max values. P values were determined by using the Kruskal-Wallis test with Dunns multiple test correction.

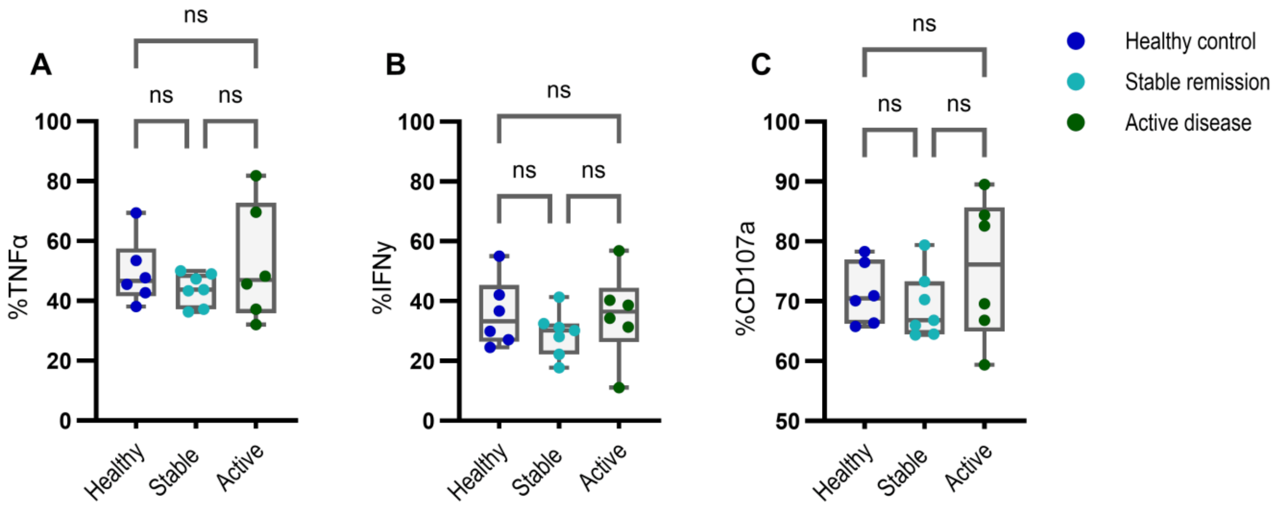

Supplementary Figure 3. In-vitro functional responses of CD56^{bright} NK cells is similar across RA disease activity states. The proportion of IL-2 stimulated CD56^{bright} NK cells subsets expressing **(A)** TNFα, **(B)** IFNγ**,** and **(C)** CD107a in response to K562 target cell interaction. Whiskers on plots represent min to max values. P values were determined by using the Kruskal-Wallis test with Dunns multiple test correction.

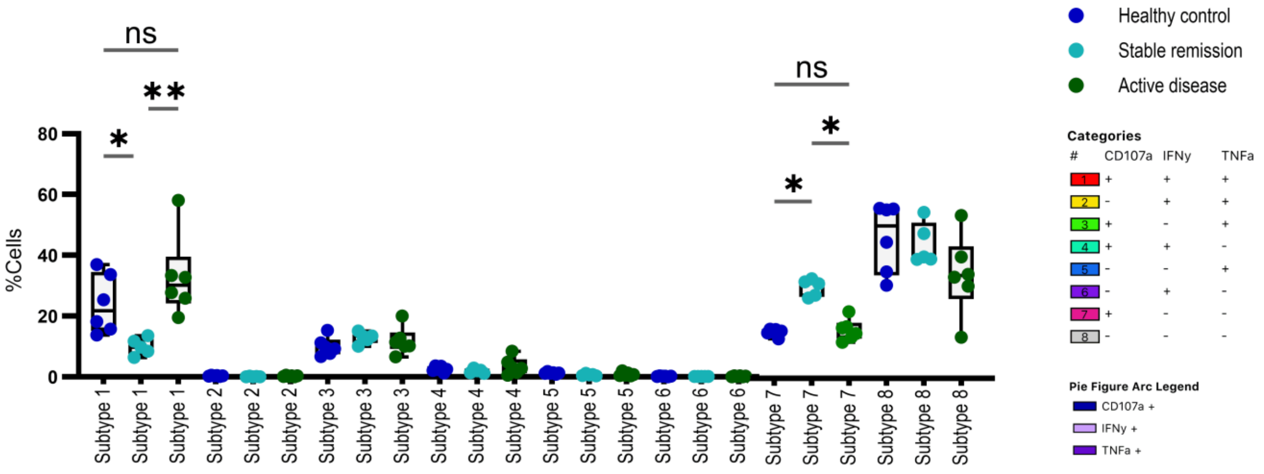

Supplementary Figure 4. SPICE analysis uncovers 8 functional NK cell subsets. IL-2 stimulated CD56^{dim} NK cells give rise to distinct functional subtypes in response to K562 target cell interaction. Whiskers on plot represent min to max values. P values were determined by using the Kruskal-Wallis test with Dunns multiple test correction. *P <0.05, **P <0.01.

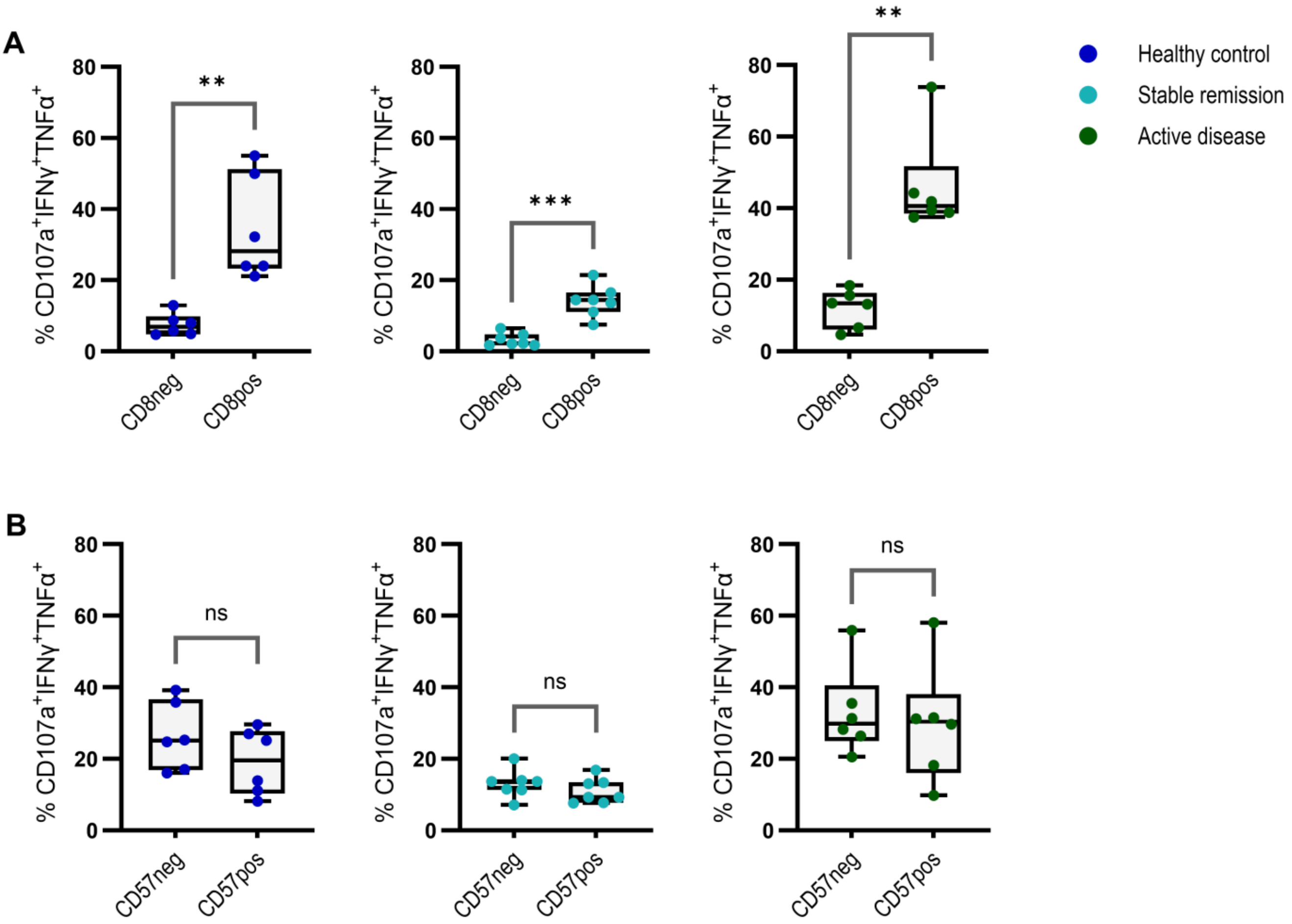

Supplementary Figure 5. Expression of CD8 is associated with enhanced polyfunctional responses. (A) Comparison of the proportion of CD107a$^+$IFNγ$^+$ TNFα$^+$ NK cell subsets between CD8$^-$ and CD8$^+$ and **(B)** CD57$^-$ and CD57$^+$ NK cells in response to IL-2 stimulation and K562 target cell interaction. Whiskers on plots represent min to max values. P values were determined by using the Mann Whitney test. **P <0.01, ***p<0.001.

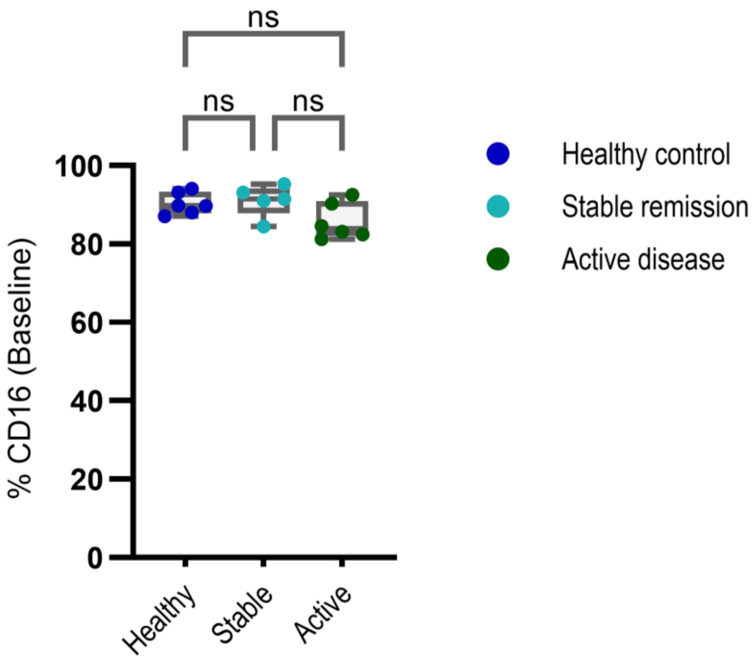

Supplementary Figure 6. Baseline levels of CD16 is consistent across groups. Baseline expression of CD16 on $CD56^{dim}CD16^{+}$ NK cells. Whiskers on plot represent min to max values. P values were determined by using the Kruskal-Wallis test with Dunns multiple test correction.

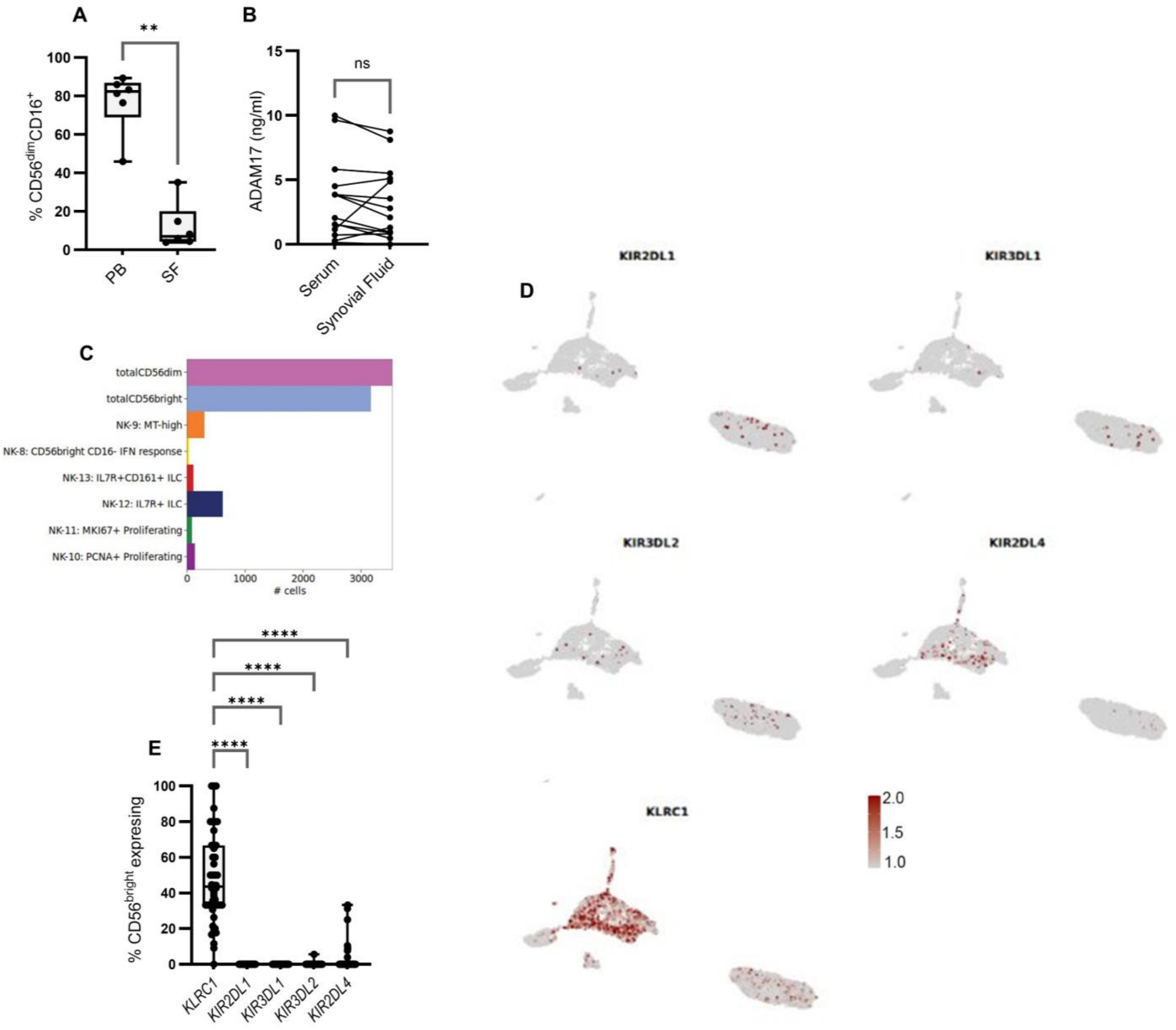

Supplementary Figure 7. NK cell phenotypes associated with stable remission are reduced from synovial tissue of active RA. (A) The proportion of CD56^{dim}CD16^{+} NK cells between peripheral blood (PB) and synovial fluid (SF), **(B)** Soluble ADAM levels in paried serum and synovial fluid, **(C)** The number of cells within 8 transcriptional clusters from single cell RNA-Seq of synovial tissue, **(D)** UMAP feature plot showing the expression of inhibitory receptors across CD56^{bright} and CD56^{dim} clusters **(E),** Comparing the proportion of CD56^{bright} NK cells expressing *KLRC1* to all cells expressing genes encoding all corresponding KIRs. Whiskers on plot represent min to max values. P values were determined by using the Mann Whitney test **(A)** and the Kruskal-Wallis test with Dunns multiple test correction **(D)** **P <0.01, ****p<0.0001.

Supplementary table 1. Mass cytometry panel information

Target	Clone	Label	Supplier	Product ID
CD11c	Bu15	147Sm	Standard Biotools	3147008B
CD16	3G8	209Bi	Standard Biotools	3209002B
CD25 (IL-2R)	2A3	169Tm	Standard Biotools	3169003B
CD28	CD28.2	160Gd	Standard Biotools	3160003B
CD38	HIT2	144Nd	Standard Biotools	3144014B
CD45	HI30	89Y	Standard Biotools	3089003B
CD45RA	HI100	155Gd	Standard Biotools	3155011B
CD56 (NCAM)	CMSSB	176Yb	Standard Biotools	3176003B
CD57	HCD57	172Yb	Standard Biotools	3172009B
CD123 (IL-3R)	6H6	151Eu	Standard Biotools	3151001B
CD127 (IL-7Ra)	A019D5	149Sm	Standard Biotools	3149011B
CD161	HP-3G10	159Tb	Standard Biotools	3159004B
CD196 (CCR6)	11A9	141Pr	Standard Biotools	3141014A
CD197 (CCR7)	G043H7	167Er	Standard Biotools	3167009A
CD183 (CXCR3)	G025H7	163Dy	Standard Biotools	3163004B
CD185/CXCR5	51505	164Dy	Standard Biotools	3164016B
HLA-DR	L243	173Yb	Standard Biotools	3173005B
IgD	IA6-2	146Nd	Standard Biotools	3146005B
CD194/CCR4	L291H4	158Gd	Standard Biotools	3158032A
CD95/Fas	DX2	152Sm	Standard Biotools	3152017B
CD24	ML5	166Er	Standard Biotools	3166007B
TCR Va7.2	3C10	153Eu	Standard Biotools	3153024B
CD278/ICOS	C398.4A	143Nd	Standard Biotools	3143025B
CD80 (B7-1)	2D10.4	162Dy	Standard Biotools	3162010B
CD86	IT2.2	156Gd	Standard Biotools	3156008B
CD279 (PD-1)	EH12.2H7	175Lu	Standard Biotools	3175008B
CD40	5C3	165Ho	Standard Biotools	3165005B
CD274 (PD-L1)	29E.2A3	148Nd	Standard Biotools	3148017B
CD152 (CTLA-4)	14D3	161Dy	Standard Biotools	3161004B
CD14	M5E2	171yb	Biolegend	301843
CD3	UCHT1	112Cd	Biolegend	300443
CD4	RPA-T4	114Cd	Biolegend	300541
CD8	SK1	113Cd	Biolegend	344727
CD20	2H7	116Cd	Biolegend	302343
CD19	HIB19	111Cd	Biolegend	302247

Supplementary table 2. Validation sample spectral flow panel

Target	Label	Clone	Supplier	Product ID
CD16	BUV395	3G8	BD	563784
CD8	BUV563	PRA-T8	BD	612915
FVS-455UV	eFlour455UV	N/A	Thermofisher	65-0868-14
CD226	BUV737	118A	BD	752663
Streptavidin	PerCP-Cy5.5	N/A	Biolegend	405214
NKp46	BV421	9E2	Biolegend	331913
CD57	BV510	QA17A04	Biolegend	393313
CD117	104D2	BV650	Biolegend	313221
CD56	BV785	5.11H11	Biolegend	362550
KIR2DL1	FITC	HP-MA4	Biolegend	339503
CRTH2	BM16	PE-Dazzle	Biolegend	350125
CD127	A019D5	PE-Cy5	Thermofisher	605-210
CD45	PerCP	2D1	Biolegend	368506
NKG2A	APC/Fire 750	S19004C	Biolegend	375115
NKG2D	APC	1D11	Biolegend	320807

* CD127, CD177 and CRTH2 was included to exclude any innate lymphoid cells (ILC) which may be present
* Streptavidin was included to detect any remaining biotinylated CD3 and CD19 which was not depleted by the Dynabead enrichment

Supplementary table 3. Functional analysis spectral flow panel

Target	Label	Clone	Supplier	Product ID
CD16	BUV395	3G8	BD	563784
FVS-455UV	eFlour455UV	N/A	Thermofisher	65-0868-14
CD8	AF700	RPA-T8	Biolegend	301027
IFNy	BUV737	4S.B3	BD	612845
NKp46	BV421	9E2	Biolegend	331913
CD57	BV510	QA17A04	Biolegend	393313
TNFa	BV650	MAb11	Biolegend	503937
CD56	BV785	5.11H11	Biolegend	362550
KIR2DL1	FITC	HP-MA4	Biolegend	339503
CD69	PE-Cy5	FN50	Biolegend	310907
CD107a	PE-Cy7	HA43	Biolegend	328617
CD45	PerCP	2D1	Biolegend	368506
NKG2A	APC/Fire 750	S19004C	Biolegend	375115

Supplementary table 4. Validation sample set information

	Healthy controls (n = 6)	Stable remission (n = 14)	Intermittent remission (n = 8)	Active Disease (n = 8)
**Demographics**				
Age in years, mean(SD)	52.50 (11.33)	62.86 (11.54)	66.22 (7.98)	60.88 (9.03)
Female/male, n (%)	5 (83) / 1 (17)	10 (71) / 4 (29)	6 (75) / 2 (15)	5 (63) / 3 (37)

	Stable remission	Intermittent remission	Active Disease
**Clinical parameters**			
Disease duration in years, mean(SD)	3.6 (2.9)	3.3 (2.7)	3.5 (2.8)
DAS28, mean(SD)	1.40 (0.44)	2.09 (0.41)	4.12 (0.5)
VASP[A] in mm, mean(SD)	5.07 (6.50)	35.56 (29.4)	38.50 (30.6)
FACIT-F[B], mean (SD)	27.50 (25.21)	41.22 (28.60)	45.13 (22.28)
HAQ[C], mean (SD)	0.20 (0.31)	0.76 (0.75)	0.72 (0.52)
Swollen joint count, mean (SD)	0.58 (0.85)	1.22 (0.83)	1.25 (0.73)
Tender joint count, mean (SD)	0 (0)	0.11 (0.33)	5.88 (4.7)
Physician global assessment, mean (SD)	1.4 (5.3)	15.63 (8.2)	27.50 (13.89)
Patient global assessment, mean (SD)	9.0 (8.1)	35.78 (26.3)	46.38 (25.1)
**Laboratory parameters**			
ESR (mm/hr), mean (SD)	6 (3.1)	11 (8.2)	15.88 (8.4)
CRP (mg/dl), mean (SD)	5 (1.3)	4.5 (1.0)	7.2 (4.6)
**Treatment information**			
Methotrexate, n (%)	14 (100)	8 (100)	8 (100)
Hydroxychloroquine, n (%)	3 (21)	3 (38)	2 (25)
Sulfasalazine, n (%)	2 (14)	1 (13)	0 (0)
Leflunomide, n (%)	0 (0)	1 (13)	1 (13)

Demographics, clinical characteristics and treatment information relating to samples analysed in Figure 1E-G, Figure 2 and Figure 3

[A] Visual analogue scale for pain, [B] Functional Assessment of Chronic Illness Therapy- Fatigue, [C] Health assessment questionnaire

Supplementary table 5. Functional studies sample information

	Healthy controls (n = 6)	Stable remission (n = 7)	Active Disease (n = 6)
Age in years, mean(SD)	52.50 (11.33)	60 (13.63)	52 (18.63)
Female/male, n (%)	5 (83) / 1 (17)	4 (57) / 3 (43)	5 (83) / 1 (17)
DAS28, mean(SD)	N/A	1.28 (0.55)	4.92 (0.53)

Information relating to samples analysed in Figure 4 and Figure 5

Supplementary table 6. Paired PBMC/SFMC sample information

	Active Disease (n = 6)
Age in years, mean(SD)	57 (21.96)
Female/male, n (%)	6 (100) / 0 (0)
DAS28, mean(SD)	4.5 (1.3)

Information relating to samples analysed in Figure 6
